# Editorial: Understanding and Modulating Bone and Cartilage Cell Fate for Regenerative Medicine

**DOI:** 10.3389/fbioe.2019.00008

**Published:** 2019-01-28

**Authors:** Roberto Narcisi, Eric Farrell

**Affiliations:** ^1^Department of Orthopedics, Erasmus MC, University Medical Center, Rotterdam, Netherlands; ^2^Department of Oral and Maxillofacial Surgery, Special Dental Care and Orthodontics, Erasmus MC, University Medical Center, Rotterdam, Netherlands

**Keywords:** endochondral ossification, cartilage, bone, cell fate, mechanical loading, osteoarthritis

With this research topic we provide an overview of the main tools regenerative medicine and stem cells research have to better understand and modulate bone and cartilage cell fate, both during natural healing processes and during the development of joint pathologies. Moreover, the contribution to the research topic with original research articles allow a further exploration toward the most advanced research in the field.

What is the role of mechanical loading to determine cell fate during bone development? How can we modulate biomaterial proprieties to drive cellular differentiation of stem cells toward cartilage and bone? What we can learn about bone and cartilage cell fate by following natural healing processes and osteoarthritis development? This Research Topic explores these and other crucial questions in the field of bone and cartilage regeneration, with the intention to trigger the development of new research lines and further increase of knowledge.

Scaffold manufacturing and specific types and regimes of mechanical stimulation are known to be essential for supporting and promoting cellular differentiation. Specifically, Hendrikson et al. show how different scaffold architectures have significant influence on stress and strain distribution, but also on the effective pore size and shape, which subsequently influence the fluid shear stress distribution.  Angelozzi et al. discuss how the use of microfibrous alginate scaffolds containing gelatin or the more innovative urinary bladder matrix (UBM) are able to stimulate dedifferentiated chondrocyte to re-acquire their natural phenotype. Mesenchymal stem/progenitor cells (MSC) are often use to recapitulate the endochondral ossification process. For this reason Carroll et al. used MSC as a model to explore the role of cyclic tensile strain during their differentiation showing that this specific mechanical stimuli can play a role in promoting both intramembranous and endochondral ossification of MSC in a context-dependent manner. Dynamic mechanical compression is also one of the most used strategies to regulate cellular phenotype. However, as highlighted by Anderson and Johnstone, the lack of standardized methods and analysis to study chondrogenic differentiation and maintenance under this mechanical regimes make the comparison between the current literature difficult.

Understanding the role and the mechanism of action of endogenous bone and cartilage repair by progenitor cells is pivotal to increase the knowledge around endogenous progenitor cell function and therefore improve the development of tissue repair strategies. Lo Sicco and Tasso provide an overview on the novel findings that impact bone fracture healing, with a particular focus on the role of inflammation, progenitor cell recruitment and their differentiation. Lozito TP's group, on the other hand, used the lizard tail regeneration model to determine the cellular origin of regenerated cartilage and muscle following tail loss, interestingly showing how cartilage cells can contribute to the regeneration of both muscle and cartilage tissue (Londono et al.).

In order to have a whole overview on endochondral fracture healing, Wong et al. reviewed the molecular pathways that (may) play a role in modulating cellular fate during endochondral fracture healing, Lesage et al. discussed the current methodologies to analyze cell differentiation during endochondral ossification using computational modeling and Javaheri et al. reviewed the interesting recent findings supporting the idea that hypertrophic chondrocytes have pluripotent capacity and may transdifferentiate into osteoblastic cells. To complete the overview on fracture healing, the group of Correa D proposed a comprehensive summary on tissue engineering strategies for fractures and bone defects, discussing not only the role of the cells but also the impact of the use of different biomaterials and grow factors to stimulate the healing process (Perez et al.).

Sometimes a pathological situation can be very useful to study physiological processes. Osteoarthritis (OA), a pathology of the diarthoroidal joints, is a diseased state where all the joint tissues are involved, leading to cartilage and bone changes. Raman et al. addressed the key inflammatory factors and the main epigenetic changes that occur in chondrocytes during OA and how we may be able to reverse them. In parallel, the group of Welting TJM discussed the current knowledge regarding the cartilage endochondral changes occurring during OA (Ripmeester et al.), overall covering most of the literature available in the field up to date. It is also interesting to observe how knowledge on the endochondral ossification process can also derive by the observation of physiological processes apparently non related to it. One great example are vascular diseases, such as atherosclerosis, where vascular calcification is observed, with many aspects of the process of endochondral ossification apparent. Leszczynska and Murphy reviewed this scenario focusing on the (circulating) cellular players contributing to form ectopic bone and cartilage during atherosclerosis.

The issues discussed in this Research Topic address several important biological aspects that determine the fate of cells in the cartilage and bone leading to the repair of damaged tissues or the onset of disease. Improving our understanding of these processes will allow us to further refine regenerative medicine based approaches to the treatment of many bone and cartilage related pathologies.

## Author Contributions

All authors listed have made a substantial, direct and intellectual contribution to the work, and approved it for publication.

### Conflict of Interest Statement

The authors declare that the research was conducted in the absence of any commercial or financial relationships that could be construed as a potential conflict of interest.

